# Auditory hedonic phenotypes in dementia: A behavioural and neuroanatomical analysis

**DOI:** 10.1016/j.cortex.2015.03.021

**Published:** 2015-06

**Authors:** Phillip D. Fletcher, Laura E. Downey, Hannah L. Golden, Camilla N. Clark, Catherine F. Slattery, Ross W. Paterson, Jonathan M. Schott, Jonathan D. Rohrer, Martin N. Rossor, Jason D. Warren

**Affiliations:** Dementia Research Centre, UCL Institute of Neurology, University College London, United Kingdom

**Keywords:** Environmental sounds, Music, Musicophilia, Reward, Affect, Alzheimer's disease, Frontotemporal dementia, Semantic dementia, Progressive aphasia, VBM

## Abstract

Patients with dementia may exhibit abnormally altered liking for environmental sounds and music but such altered auditory hedonic responses have not been studied systematically. Here we addressed this issue in a cohort of 73 patients representing major canonical dementia syndromes (behavioural variant frontotemporal dementia (bvFTD), semantic dementia (SD), progressive nonfluent aphasia (PNFA) amnestic Alzheimer's disease (AD)) using a semi-structured caregiver behavioural questionnaire and voxel-based morphometry (VBM) of patients' brain MR images. Behavioural responses signalling abnormal aversion to environmental sounds, aversion to music or heightened pleasure in music (‘musicophilia’) occurred in around half of the cohort but showed clear syndromic and genetic segregation, occurring in most patients with bvFTD but infrequently in PNFA and more commonly in association with *MAPT* than *C9orf72* mutations. Aversion to sounds was the exclusive auditory phenotype in AD whereas more complex phenotypes including musicophilia were common in bvFTD and SD. Auditory hedonic alterations correlated with grey matter loss in a common, distributed, right-lateralised network including antero-mesial temporal lobe, insula, anterior cingulate and nucleus accumbens. Our findings suggest that abnormalities of auditory hedonic processing are a significant issue in common dementias. Sounds may constitute a novel probe of brain mechanisms for emotional salience coding that are targeted by neurodegenerative disease.

## 1 Introduction

Altered emotional responsiveness to salient sensory stimuli is a key issue in neurodegenerative diseases. From a clinical perspective, emotional dysregulation is likely to contribute to a wide spectrum of symptoms that impact the lives of patients and their caregivers: examples range from abnormal responses to thermoregulatory and other basic homeostatic signals (Ahmed et al., 2014) to pathological seeking of rewarding stimuli such as food, sex or drugs (Perry et al., 2014; Whitwell et al., 2007; Woolley et al., 2007) and derangements of complex social behaviours (Clark, Downey, Golden, et al., 2014; Mahoney, Rohrer, Omar, Rossor, & Warren, 2011; Sturm et al., 2013). From a neurobiological perspective, such dysfunction illuminates critical neural mechanisms mediated by brain networks that are targeted by neurodegenerative pathologies (Zhou & Seeley, 2014). Altered emotional responses are a hallmark of diseases in the frontotemporal lobar degeneration (FTLD) spectrum, particularly in association with the syndromes led by behavioural disintegration (behavioural variant frontotemporal dementia, bvFTD) and semantic disintegration (semantic dementia, SD) (Duval et al., 2012; Hodges & Patterson, 2007; Rankin et al., 2009; Rohrer & Warren, 2010; Snowden et al., 2001). However, emotional disturbances may also be significant in other FTLD syndromes such as progressive nonfluent aphasia (PNFA) (Kumfor et al., 2011; Rohrer & Warren, 2011; Rohrer, Sauter, Scott, Rossor, & Warren, 2012) and with other neurodegenerative pathologies, notably Alzheimer's disease (AD) (Sturm et al., 2013). This phenotypic overlap reflects the involvement in these diseases of distributed fronto-temporal, parietal and subcortical circuitry previously implicated in the representation, decoding and evaluation of salient stimuli (Sescousse, Caldu, Segura, & Dreher, 2013).

In contrast to the better characterised phenotypes of language, perceptual and executive impairment, phenotypes of altered emotional responsiveness are difficult to capture using standard neuropsychological instruments and remain poorly defined in the dementias. This reflects the inherently complex organisation of emotional behaviour, which is often only partly accessible to explicit cognitive decoding and relies intimately on subjective states of emotional awareness or affect. Besides neuropsychological and psychophysical procedures, a complete characterisation of affective responses in patients with dementia requires detailed analysis of output behaviours, particularly those signalling pleasure or aversion. Furthermore, it is necessary to sample a wide range of stimuli and behaviours, as alterations of affective processing in neurodegenerative diseases may extend to categories of stimuli that lack primary biological reward value. The auditory domain is a particularly promising vehicle with which to explore affective abnormalities in these diseases, since sound encompasses a broad continuum of sensory signals ranging from the highly biologically or perceptually salient to the banal to the richly symbolic. A key example of the last is music: this essential abstract stimulus has been shown to engage reward circuitry extensively in the healthy brain (Blood & Zatorre, 2001; Blood, Zatorre, Bermudez, & Evans, 1999; Koelsch, 2014; Menon & Levitin, 2005; Salimpoor et al., 2013; Salimpoor, Zald, Zatorre, Dagher & McIntosh, 2015) and musical pleasure is associated with powerful autonomic responses (Grewe, Nagel, Kopiez, & Altenmüller, 2005). Musical pleasure is likely to depend heavily on factors such as familiarity, pattern recognition and predictability based on past experience (Koelsch, 2014; Salimpoor et al., 2015; Zatorre & Salimpoor, 2013): the integrative neural computations required are likely to be vulnerable in the dementias. Music may become the object of obsessional interest or ‘musicophilia’ in neurodegenerative syndromes (Fletcher, Downey, Witoonpanich, & Warren, 2013), and more generic abnormalities of affective responsiveness to hedonically neutral sounds also occur: for example, a substantial proportion of patients with SD exhibit increased sensitivity and aversion to everyday environmental noises (Mahoney, Rohrer, Goll, et al., 2011). As the symptom profile of SD encompasses both heightened pleasure in music and aversion to environmental sounds, it is evident that changes in auditory hedonic responsiveness produced by neurodegenerative syndromes are likely to be complex and bi-directional. Neuroanatomical correlates of these symptoms have been described in auditory cortical and subcortical pathways, antero-mesial temporal and frontal reward circuitry (Fletcher et al., 2013; Mahoney, Rohrer, Goll, et al., 2011; Mahoney, Rohrer, Omar, Rossor & Warren, 2011). More generic alterations in affective and autonomic responses to emotional sounds have been described in various dementia syndromes (Fletcher et al., in press-a, in press-b). These observations underline the potential of sound to probe brain networks that mediate affective responses and are targeted by neurodegenerative diseases. However, phenotypes of altered affective response to sound and their brain bases have not been studied systematically across dementia syndromes.

Here we addressed this issue in cohorts of patients representing major canonical syndromes of FTLD and AD. Altered hedonic responses to nonverbal sound – increased pleasure, anhedonia or aversion to environmental sounds and music – in these diseases were indexed from patients' verbal and nonverbal behaviours, as recorded using a semi-structured caregiver questionnaire. The questionnaire also recorded any alterations in patients' sweet food preferences, in order to assess hedonic responses of sounds in relation to a hedonic behaviour that is commonly affected in dementia but linked to a primary biological reward (Perry et al., 2014; Whitwell et al., 2007; Woolley et al., 2007). Structural neuroanatomical substrates of abnormal auditory hedonic responses were assessed using voxel-based morphometry (VBM) of patients' brain MR images. Based on previous clinical evidence, we hypothesised that these neurodegenerative syndromes would produce a complex of auditory hedonic abnormalities with bi-directional shifts in the valuation of particular sound categories (environmental sound and music); and that such abnormalities would be more common in the syndromes of bvFTD and SD than other neurodegenerative syndromes, and would correlate with altered sweet food preference. We further hypothesised that altered auditory hedonic responsiveness would be associated with grey matter changes in a distributed brain network including areas previously implicated in encoding the affective salience of sounds and other sensory signals (in particular, insula and anterior cingulate cortex: Zhou & Seeley, 2014), evaluating their affective meaning (in particular, antero-mesial temporal lobe: Omar et al., 2011; Hsieh, Hornberger, Piguet, & Hodges, 2012) and representing their reward value (in particular, ventral striatum: Sescousse et al., 2013).

## 2 Methods

### 2.1 Patient characteristics

A cohort of 73 patients was recruited over a three-year interval via a tertiary cognitive disorders clinic. The cohort comprised 56 patients with a syndrome of FTLD (bvFTD, n = 22; SD, n = 19; PNFA, n = 15) and 17 patients with amnestic AD. All were diagnosed by an experienced cognitive neurologist and fulfilled consensus diagnostic criteria (Dubois et al., 2007; Gorno-Tempini et al., 2011; Rascovsky et al., 2007). The syndromic diagnosis was supported in each case by detailed neuropsychological assessment (in relation to an historical age- and gender-matched cohort of 50 healthy individuals: Table 1) following a standard protocol and further corroborated by CSF or brain amyloid PET imaging findings (ratio of total tau: beta-amyloid_1-42_ levels >1 in 9/9 AD patients and <.8 in 14/14 FTLD patients, Florbetapir PET negative for amyloid deposition in 6/6 FTLD patients for whom data were available). All patients had a profile of regional brain atrophy on MRI consistent with the syndromic diagnosis; no patient had radiological evidence of significant or strategic vascular damage. Genetic screening of the patient cohort revealed 13 patients with a pathogenic mutation (seven *C9orf72*; six *MAPT*). All patients with a genetic mutation presented with bvFTD apart from one patient with a *C9orf72* expansion who presented with PNFA.

Patients with clinically relevant alterations of peripheral hearing loss were not included in the study; 12 patients with hearing loss and eight patients with tinnitus were excluded on this basis. Participants underwent screening of peripheral hearing function using a previously described adapted audiometry procedure (Golden et al., 2015).

All participants gave written informed consent to be involved in the study, which was approved by the local institutional ethics committee in accordance with the Declaration of Helsinki.

### 2.2 Analysis of hedonic symptoms

Patient caregivers were asked to complete a questionnaire detailing any symptoms suggesting alterations in the pleasure that patients derived from nonverbal sounds (environmental sounds and/or music) (see Table S1 in Supplementary Material on-line). Altered auditory hedonic responses were classified generally as increased or decreased liking for environmental sounds and increased or decreased liking for music. Before completing the questionnaire, caregivers were given examples of behaviours that might signal altered liking for sounds relative to the patient's premorbid behaviour (such as expressed liking or aversion for the sound, seeking or avoidance of the sound and/or amount of time spent listening to music). The caregiver questionnaire also recorded any alteration in patients' sweet food preference.

Patient subgroups with and without hedonic symptoms and healthy controls were compared using linear regression and proportions exhibiting symptoms were compared using Pearson's chi-square test. Relations of auditory hedonic symptoms to disease duration and severity (MMSE score) and with altered sweet food preference were assessed using Pearson's correlation tests. A threshold *p* < .05 was accepted as the criterion for a statistically significant difference in all comparisons.

### 2.3 Brain image acquisition and VBM

At the time of questionnaire data collection each patient underwent volumetric brain MRI on a 3.0 T Siemens scanner (Siemens, Erlangen, Germany) using a 32 channel phased array head coil. A sagittal 3-D magnetization prepared rapid gradient echo T1 weighted volumetric MRI (echo time/repetition time/inversion time 2.9/2200/900 ms, dimensions of 256 × 256 × 208, voxel size of 1.1 × 1.1 × 1.1 mm) was acquired. In all cases, volumetric scans were assessed visually in all planes to ensure adequate coverage and to exclude artefacts or significant motion.

Pre-processing of patient brain MR images for VBM was performed using New Segment (Ashburner & Friston, 2005) the DARTEL (Ashburner, 2007) toolbox of SPM8 (www.fil.ion.ucl.ac.uk/spm) running under Matlab7.0®. Segmentation, normalisation and modulation of grey and white matter images were performed using default parameter settings. Images were smoothed using a Gaussian filter with full-width-half-maximum 6 mm. In order to adjust for individual differences in global grey matter volume during subsequent analysis, total intracranial volume was calculated for each participant by summing grey matter, white matter and cerebrospinal fluid volumes following segmentation of all three tissue classes. A study-specific group mean template brain image was created by warping all native space whole-brain images to the final DARTEL template and calculating the average of the warped brain images.

Using linear regression, voxel intensity (grey matter volume) was modelled separately over the combined FTLD cohort and within the AD cohort as a function of the presence of altered liking for any sounds, and altered liking for music or environmental sounds in isolation. Patient age, total intracranial volume, disease duration and (in the FTLD analysis) syndromic group were included as covariates of no interest. Anatomical small volumes of interest based on the prior anatomical hypotheses were created to cover key regions in each cerebral hemisphere previously implicated in hedonic processing of sounds and other sensory stimuli (Sescousse et al., 2013): these regions comprised antero-mesial temporal lobe (cortex anterior to Heschl's gyrus, amygdala and hippocampus), insula and anterior cingulate cortex and ventral striatum (nucleus accumbens, caudate and putamen). Regions were customised from the Oxford/Harvard brain maps in FSLview v3.1 (Desikan et al., 2006; Jenkinson, Beckmann, Behrens, Woolrich, & Smith, 2012) to fit the mean brain template. To help protect against voxel drop-out due to potentially marked local regional atrophy in particular scans, a customised explicit brain mask was applied based on a specified ‘consensus’ voxel threshold intensity criterion (Ridgway et al., 2009) whereby a voxel was included in the analysis if grey matter intensity at that voxel was >.1 in >70% of participants (rather than in all participants, as with the default SPM8 mask). Statistical parametric maps of regional grey matter volume correlating with presence or absence of symptoms were examined at threshold *p* < .05 after family-wise error (FWE) correction for multiple voxel-wise *t*-tests over the whole brain and after small volume correction within pre-specified anatomical regions.

## 3 Results

### 3.1 General participant characteristics

Demographic, clinical and general neuropsychological characteristics of the patient cohort are summarised in Table 1. Participant subgroups (FTLD versus AD and within each disease group, subgroups with and without altered sound pleasure) did not differ in age, gender, years of education, disease duration or overall severity (based on MMSE score). Peripheral hearing function based on audiometric screening did not differ significantly (*p* > .05) between groups. On general neuropsychological assessment the FTLD subgroup with auditory hedonic symptoms performed significantly worse on the recognition memory test for faces than the FTLD subgroup without such symptoms; there were no other significant neuropsychological differences between disease subgroups with and without auditory hedonic symptoms.

### 3.2 Characteristics of hedonic symptoms

**Fig. 1:**
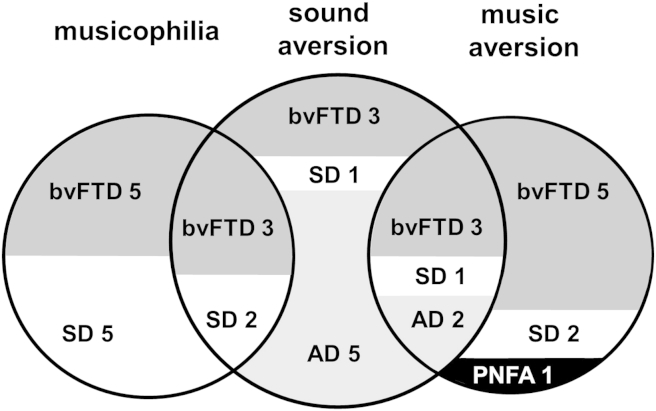
Diagrammatic breakdown of auditory hedonic symptoms across the patient cohort. Case numbers in each symptom category are indicated. Key: AD, syndrome of Alzheimer's disease led by decline in episodic memory (light grey); bvFTD, behavioural variant of frontotemporal dementia (dark grey); PNFA, progressive nonfluent aphasia (black); SD, semantic dementia (white).

Symptoms of altered auditory hedonic valuation occurred in a substantial proportion of patients in both the FTLD cohort (31/56 cases, 55%) and the AD cohort (7/17 cases, 41%), with comparable overall frequency in each disease (*p* = .31). The breakdown of auditory hedonic symptoms by diseases and syndromes is schematised in Fig. 1. Within the FTLD cohort, symptoms were significantly more common in the bvFTD group (19/22 cases, 86%) than the SD group (11/19 cases, 58%) (*p* = .04) and in both the bvFTD and SD groups than the PNFA group (1/15 cases, 7%) (*p* < .01). Altered liking for environmental sounds and for music were each exhibited by patients in both the FTLD and AD cohorts, however the relative frequency and directionality of these symptoms varied between diseases: patients with FTLD who developed auditory hedonic symptoms variously exhibited decreased liking for environmental sounds (13/31 cases, 42%), decreased liking for music (12/31 cases, 39%) or increased liking for music (‘musicophilia’; 15/31 cases, 48%) alone or in combination, whereas those patients with AD who developed hedonic symptoms uniformly exhibited decreased liking for sounds (environmental sounds in 7/7 cases; music additionally in 2/7 cases, 29%). Only one patient in the entire study cohort experienced abnormally increased liking for neutral environmental sounds: this patient with a syndromic diagnosis of SD derived pleasure from certain mechanical sounds such as a hair-dryer as well as exhibiting a heightened emotional response to music.

Caregiver questionnaire reports indicated a diverse phenomenology of altered auditory hedonic responses in individual patients (representative extracts for individual patients are in Table S2 in Supplementary Material on-line). Typically patients with reduced liking for environmental sounds were described by caregivers as having become unusually sensitive to the relevant sound since onset of their illness; exposure to certain environmental noises (particularly those with higher pitch or penetrating timbre such as children's voices) would provoke expressions of distress in these patients and they would take sometimes elaborate steps to avoid such sounds, even in situations where these would previously have been regarded as unobtrusive or banal. Patients with reduced liking for music were described as exhibiting a wider repertoire of responses, ranging from indifference (loss of previous interest and enjoyment) to active avoidance, distress or irritation. In order to capture this dynamic component of reduced liking, henceforth we refer to environmental sound and music ‘aversion’. Conversely, patients with increased liking for music [in line with previous descriptions of musicophilia: (Fletcher et al., 2013)] exhibited music craving or seeking, often demanding to listen to a narrow repertoire of songs for up to many hours each day but sometimes also engaging in more organised behaviours such as taking up a musical instrument or buying music equipment.

Within the FTLD subgroup with altered auditory hedonic responses, patients with bvFTD and SD were comparably likely to develop environmental sound aversion (bvFTD 9/19 cases, 47%; SD 4/11 cases, 36%; *p* = .56); there was the impression of an over-representation in the SD group of patients with musicophilia (bvFTD 8/19 cases, 42%; SD 7/11 cases, 64%) versus music aversion (bvFTD 8/19 cases, 42%; SD 3/11 cases, 27%), however this apparent disproportion did not achieve statistical significance when the SD and bvFTD groups were compared directly (*p* = .42). Musicophilia was accompanied by environmental sound aversion in a substantial minority of patients with FTLD (bvFTD 3/19 cases, 16%; SD 2/11 cases, 18%); a comparable proportion of patients (6/38 cases, 16% of the combined cohort) exhibited aversion to both sound categories. The single patient with PNFA who developed auditory hedonic symptoms exhibited music aversion. Genetic FTLD subtype influenced the development of auditory hedonic alterations: symptoms were significantly more common in the *MAPT* mutation group (6/6 cases) than the *C9orf72* mutation group (3/7 cases) (*p* = .03). Patients in both these genetic subgroups tended to exhibit aversion to sounds; musicophilia was reported only in isolated cases in each subgroup (in each case accompanied by environmental sound aversion).

Compared with auditory hedonic symptoms, pathological sweet–tooth developed in a similar proportion of the FTLD cohort overall (35/56 cases, 63%) and in association with bvFTD (21/22 cases, 95%) and SD (9/19 cases, 47%). Pathological sweet tooth was relatively more frequent than auditory hedonic alterations in association with PNFA (5/15 cases, 33%) but less frequent than auditory hedonic alterations in AD (5/17 cases, 29%). Development of pathological sweet tooth was significantly correlated with development of any auditory hedonic symptoms in both the FTLD and AD groups (*p* = .02 and *p* = .001, respectively) but not more specifically with a particular auditory hedonic phenotype. Moreover, increased liking for sweet foods was frequently coupled with reduced liking for sounds (15/31 patients with auditory symptoms in the FTLD cohort and 4/7 patients with auditory symptoms in the AD cohort). Development of auditory hedonic symptoms or pathological sweet tooth were not significantly correlated with disease duration or severity (MMSE score), in either FTLD or AD.

### 3.3 Neuroanatomical associations

**Fig. 2:**
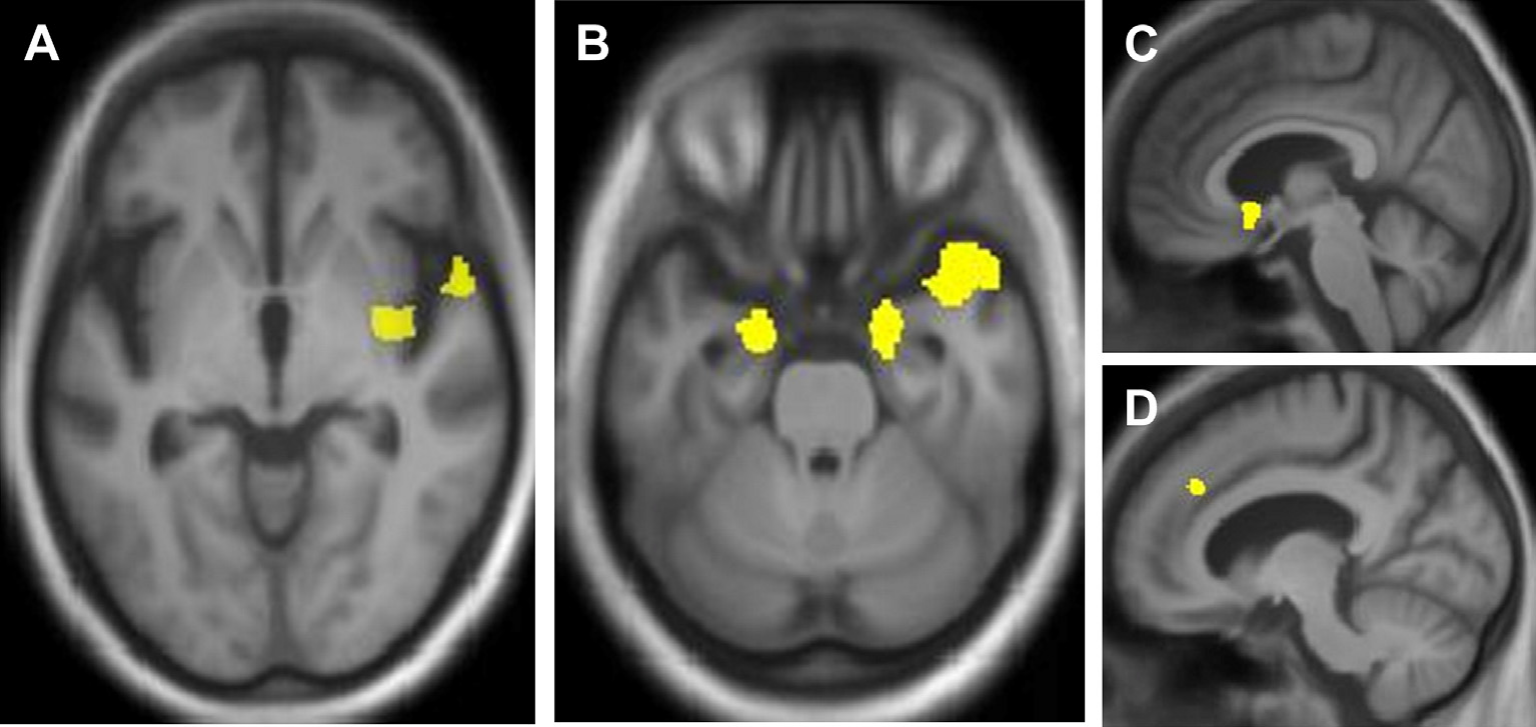
Statistical parametric maps (SPMs) showing regional grey matter atrophy significantly associated with: **A**, any auditory hedonic symptoms in the combined FTLD cohort, centred on right anterior temporal lobe, insula and putamen; **B**, **C**, environmental sound aversion in the combined FTLD cohort, including anterior temporal cortex, amygdala and nucleus accumbens; **D**, environmental sound aversion in the AD cohort, in anterior cingulate cortex. The neuroanatomical associations of music aversion in the FTLD cohort (not shown) comprised a similar distributed fronto-temporal network (see Table 2). SPMs are thresholded at *p* < .05 after small volume correction for multiple voxel-wise comparisons in pre-specified small anatomical volumes of interest and displayed on sections of a group mean T1-weighted MR brain template image in MNI standard space; the right hemisphere is shown on the right of the axial sections. AD, Alzheimer's disease; FTLD, frontotemporal lobar degeneration.

Regional grey matter correlates of auditory hedonic symptoms from the VBM analysis are summarised in Table 2 and statistical parametric maps are shown in Fig. 2.

At the most stringent statistical criterion (*p* < .05_FWE_ corrected over the whole brain volume), within the combined FTLD cohort the presence of any auditory hedonic symptoms was associated with grey matter loss in right temporal pole and anterior superior temporal cortex, extending into mid and posterior insula and putamen; while the presence of environmental sound aversion alone was also associated with grey matter loss in right anterior temporal lobe and insula, extending to include right amygdala, hippocampus, entorhinal and parahippocampal cortex.

No other grey matter associations of auditory hedonic symptoms were identified at whole brain level. However, further neuroanatomical associations were identified in the FTLD cohort at significance threshold *p* < .05_FWE_ corrected within the anatomical regions specified by our prior hypotheses. Using this criterion, environmental sound aversion was associated with additional grey matter loss in left amygdala and nucleus accumbens. Music aversion was associated with grey matter loss in an overlapping network including right anterior temporal cortex, entorhinal cortex, hippocampus and amygdala and bilateral mid and posterior insula. No neuroanatomical associations of musicophilia were identified at the prescribed significance threshold; however, a post hoc analysis at a more lenient threshold (*p* < .001 uncorrected over the whole brain) revealed relative preservation of grey matter in right hippocampus (MNI peak coordinates [38 −1 −28], z-score 3.80) in association with musicophilia.

In the AD cohort, the presence of environmental sound aversion was associated with grey matter loss in anterior cingulate cortex at significance threshold *p* < .05_FWE_ corrected within the pre-specified anatomical region of interest.

## 4 Discussion

Here we have presented evidence for phenotypes of abnormal hedonic processing in canonical dementia syndromes. Behavioural responses signalling abnormal aversion to environmental sounds, aversion to music or heightened pleasure in music (‘musicophilia’) were reported by caregivers in around half of patients with FTLD and AD overall. However, auditory hedonic symptoms showed clear syndromic and genetic segregation, occurring in most patients with bvFTD but infrequently in PNFA and more commonly in association with *MAPT* than *C9orf72* mutations. Aversion to environmental sounds and music occurred most commonly in the patient cohort overall and was the exclusive auditory phenotype in the AD group, whereas musicophilia was the single most frequent auditory hedonic symptom in the FTLD cohort, particularly in association with SD. Complex conjoined or bivalent auditory phenotypes were frequent, particularly in the FTLD cohort. However, increased liking for environmental sounds (reported in just one patient) appears to be an uncommon phenotype. While auditory hedonic symptoms were generally correlated with development of pathological sweet tooth, individual patients not uncommonly showed dissociated hedonic profiles and abnormal liking for sweet foods was frequently accompanied by aversion to sounds. The phenotypic complexity of auditory hedonic alterations in the present cohort was underpinned by a neuroanatomical profile of grey matter loss in a common, distributed, right-lateralised network including antero-mesial temporal lobe, insula, anterior cingulate and nucleus accumbens.

These findings substantiate and extend previous evidence for altered hedonic processing of environmental sounds and music in dementia diseases, notably within the FTLD spectrum (Boeve & Geda, 2001; Fletcher et al., 2013, 2015a; Geroldi et al., 2000; Mahoney, Rohrer, Goll, et al., 2011). These auditory hedonic symptoms are representative of a broader spectrum of hedonic alterations directed variously to biologically rewarding stimuli such as food, sex and drugs (Mendez & Shapira, 2013; Perry et al., 2014; Whitwell et al., 2007; Woolley et al., 2007), secondary reinforcers such as money (Perry, Sturm, Wood, Miller, & Kramer, 2013) and abstract entities such as colours (Chan et al., 2009). The hedonic profile of AD is less well characterised than for FTLD. However, the finding of prominent sound aversion in the present AD cohort corroborates other work demonstrating enhanced sensitivity to punishments and social distress (Perry et al., 2014; Sturm et al., 2013) as well as emotional sounds (Fletcher et al., 2015a, 2015b) in AD. The neuroanatomical correlates of auditory hedonic symptoms across the present patient cohort are in line with a body of previous functional neuroimaging work implicating a temporo-insulo-striatal network in the processing of hedonically laden stimuli with potential reward (or punishment) value (Koelsch, 2014; Lawson et al., 2014; Pessiglione, Seymour, Flandin, Dolan, & Frith, 2006; Salimpoor et al., 2013; Salimpoor et al., 2015; Sescousse et al., 2013; Whitwell et al., 2007; Woolley et al., 2007). Particular components of this network are likely to decode, interpret and evaluate emotionally salient sensory signals (Dreher, 2013; Hsieh et al., 2012; Omar et al., 2011; Sescousse et al., 2013; Zhou & Seeley, 2014). Although the present study was not equipped to analyse these processes in detail, certain inferences can be made based both on previous functional neuroanatomical data in the healthy brain and previous evidence in these diseases. Fronto-insular and anterior cingulate cortices anchor a salience processing network that is core to the pathogenesis of bvFTD and functionally implicated in a number of other neurodegenerative syndromes, including AD (Seeley, Crawford, Zhou, Miller, & Greicius, 2009; Sturm et al., 2013; Zhou & Seeley, 2014). Information about salient signals may be routed via partly parallel pathways to amygdala and mesial temporal lobe structures, anterior temporal cortex and striatal and other subcortical regions (Sturm et al., 2013; Zhou & Seeley, 2014). The amygdala is heavily affected in FTLD and likely to process intensity, arousal potential and novelty of affective signals (Metereau & Dreher, 2013; Sescousse et al., 2013; Small et al., 2003). Anterior temporal cortex is a hub zone for appraising the significance and relevance of sensory signals (Mitterschiffthaler, Fu, Dalton, Andrew, & Williams, 2007) and targeted by a number of neurodegenerative pathologies: this region integrates multimodal sensory object knowledge with stored autobiographical (and especially, emotionally salient) experience, homeostatic status and reward potential via strong interconnections with insula, anterior cingulate and striatum (Olson, Plotzker, & Ezzyat, 2007). Involvement of anterior temporal cortex has been linked to impaired vocal and musical emotion recognition and more complex derangements of social signal processing in dementias (Hsieh et al., 2012; Irish, Hodges, & Piguet, 2014; Omar et al., 2011; Rohrer et al., 2012). The striatum and particularly nucleus accumbens are affected early in FTLD (Halabi et al., 2013; Moller et al., 2015); these structures code reward intensity and prediction error (Blood & Zatorre, 2001; Metereau & Dreher, 2013) and modulate activity in effector circuits that govern appetitive and avoidance behaviours (Zhou & Seeley, 2014).

Environmental sound and music aversion here had closely overlapping neuroanatomical correlates, implicating shared neural circuitry in the altered hedonic valuation of these very different auditory phenomena. Moreover, these neuroanatomical correlates were remote from more posterior and lateral temporal cortices representing auditory perceptual features. While we did not assess sound perception or recognition directly, it is plausible that these processes dissociate from the hedonic processing of sounds in patients with dementia: preserved emotional responses to music despite impaired melody perception are well attested (Matthews, Chang, De May, Engstrom, & Miller, 2009) and it is further noteworthy that auditory hedonic symptoms were uncommon in the present PNFA cohort despite previously documented auditory perceptual deficits in this syndromic group (Goll et al., 2010, 2011). Previous work further suggests that particular categories of stimuli (or reinforcement associated with those stimuli) may have partly separable brain substrates (Mas-Herrero, Zatorre, Rodriguez-Fornells, & Marco-Pallares, 2014; Metereau & Dreher, 2013; Omar et al., 2011; Sescousse et al., 2013). However, any such differentiation need not be based simply on anatomical separation per se; it might inhere in profiles of connectivity within the core network that processes hedonic value. Modulation of connection strength between nucleus accumbens, amygdala and other cortical and striatal regions has been shown to underpin reward and aversive learning in response to music and a range of other hedonically-weighted stimuli (Camara, Rodriguez-Fornells, & Munte, 2008; Cauda et al., 2011; Costa, Lang, Sabatinelli, Versace, & Bradley, 2010; Klucken et al., 2014; Menon & Levitin, 2005; Salimpoor et al., 2013), and altered connectivity between these brain regions has been linked to specific auditory and other behavioural phenotypes (Ma et al., 2010; Maudoux et al., 2012; White et al., 2013). Although the present study leaves this possibility unresolved, modulation of network connectivity might explain the differential shifts in hedonic value assigned to music and environmental sounds within the patient cohort (including bivalent shifts in individual patients) despite involvement of similar network elements. In this regard, it is of interest that hippocampal grey matter change was correlated here both with sound aversion and musicophilia: with the caveat that the present musicophilia correlate was identified at a relaxed significance criterion, this is consistent with previous evidence for relative hippocampal sparing in association with musicophilia in FTLD (Fletcher et al., 2013) and with other evidence implicating hippocampus in processing musical emotion (Fruhholz et al., 2014; Koelsch, 2014). Hippocampal mechanisms may be particularly relevant to the processing of dynamic stimuli such as sounds, by engaging temporal, contextual and mnestic processes (Frühholz, Trost, & Grandjean, 2014; Kraus & Canlon, 2012). Connectivity within brain circuits mediating hedonic processing is determined in part by genotype (Dreher, 2013; Klucken et al., 2014): this suggests a possible molecular basis for the differential phenotypic effects of *MAPT* and *C9orf72* mutations in this study, though any molecular specificity is likely to have been modulated by the topography of brain damage associated with these mutations, *MAPT* mutations particularly targeting the antero-mesial temporal lobes (Rohrer & Warren, 2011).

The present evidence for auditory hedonic phenotypes in dementia aligns these neurodegenerative diseases with a broader spectrum of disorders characterised by abnormal hedonic processing of sounds. The best known of these is tinnitus, in which auditory perceptual dysfunction often has a prominent component of emotional distress underpinned by reorganised neural networks (Kraus & Canlon, 2012; Maudoux et al., 2012; Mahoney, Rohrer, Goll, et al., 2011). Focal brain lesions and seizures may be associated with various selective alterations of musical hedonic experience and behaviour, encompassing musical anhedonia (Griffiths, Warren, Dean, & Howard, 2004; Satoh, Nakase, Nagata, & Tomimoto, 2011), indifference to dissonance (Gosselin et al., 2006), music craving (Rohrer, Smith, & Warren, 2006) and shifts in musical taste (Sellal et al., 2003), as well as fixations on musical analogues such as rhyme (Woollacott et al., 2014). More commonly, phenotypic variations such as absent or attenuated pleasure in music (musical anhedonia: Mas-Herrero et al., 2014) and strong aversive responses to particular environmental sounds (misophonia: Edelstein, Brang, Rouw, & Ramachandran, 2013; Schröder, Vulink, & Denys, 2011; Jastreboff & Jastreboff, 2015) may occur in isolation on a developmental basis in normal individuals and have been proposed to reflect specific patterns of connectivity between auditory, limbic and autonomic brain networks. Considered together, this evidence suggests that the auditory hedonic phenotypes of dementia may reflect intrinsic patterns of brain network activity that are released as the regulatory controls on those networks are damaged by the neurodegenerative process, perhaps analogous to mechanisms proposed to underpin hypersexuality (Mendez & Shapira, 2013) and hyperphagia (Perry & Kramer, 2015; Woolley et al., 2007). In particular, damage to anterior temporal mechanisms for appraising the context and significance of sounds might amplify any underlying propensity to label inherently innocuous auditory stimuli as inappropriately aversive or pleasurable (hypochondriasis in patients with right temporal lobe atrophy may reflect a similar process operating on somatic signals: Chan et al., 2009). Among those patients exhibiting auditory hedonic symptoms in the present study, environmental sounds provoked almost exclusively aversive responses that contrasted with more variably valenced responses to music: we speculate that this apparent polarisation may reflect the preeminent neurobiological roles these sound categories may once have served. From an evolutionary perspective, the most salient (and biologically invested) environmental noises are likely to have been warning signals, whereas proto-musical sounds may have been employed more ambiguously in emotional communication (Clark, Downey, & Warren, 2015; Fletcher et al., in press-b).

This study has several limitations that suggest directions for future work. Group sizes were relatively small; particularly in the case of uncommon genetic mutations, the present findings should be substantiated prospectively in larger cohorts including presymptomatic individuals, in order to assess the evolution of development of these phenotypes over the course of disease. Ultimately, histopathological correlation will be required. Brain mechanisms of deranged auditory hedonic processing will only be fully defined using a multimodal approach. Connectivity-based (functional and tractographic) neuroimaging techniques will be required in order to capture alterations in neural network integrity and activity profiles. Autonomic techniques will be required in order to track phenotypic changes that are predicted to occur in response to emotionally salient sounds such as music (Grewe et al., 2005) but which may not be accessible to explicit behavioural coding in dementia syndromes (Fletcher et al., in press-b). Sounds should be assessed in parallel to other hedonically laden stimuli: this will be particularly critical to identify specific phenotypes that may differentiate stimuli arising in the world at large from the more abstract stimuli embodied in music. It will be of particular interest to assess the extent to which environmental sound aversion developing in association with neurodegenerative disease shares phenotypic features with developmental misophonia: misophonia appears to signify a diverse spectrum of phenotypes of altered sound tolerance with relatively specific acoustic, behavioural and perhaps also physiological or functional neuroanatomical signatures (Edelstein et al., 2013; Jastreboff & Jastreboff, 2015; Schroder et al., 2013) that might mutually inform the study of neurodegenerative auditory phenotypes. There is presently interest in the potential use of music as a therapeutic tool in dementia (Hammar, Emami, Engström, & Götell, 2011; Vink, Birks, Bruinsma, & Scholten, 2004); the data presented here give grounds for a more general, systematic and principled re-examination of sound-based therapies in these diseases. Such therapies might be framed as environmental modification or listening retraining, and should be informed by psychoacoustic data and aligned with work in other clinical populations (Cavanna, 2014; Jastreboff & Jastreboff, 2015). Taking the limitations on the present study into account, our findings suggest that abnormalities of auditory hedonic processing are a significant issue in common dementias and warrant further attention. Sounds may constitute a novel probe of brain mechanisms for emotional salience coding that are targeted by neurodegenerative disease.

## Figures and Tables

**Table 1:** General demographic and neuropsychological data for patient subgroups with and without auditory hedonic symptoms.

	FTLD		AD		Healthy controls*
	Auditory hedonic	No auditory hedonic	Auditory hedonic	No auditory hedonic	
**General**					
No. (F:M)	31 (9:22)^†^	25 (15:10)	7 (2:5)^††^	10 (5:5)	50 (23:27)
Syndrome: bvFTD/SD/PNFA	19/11/1	3/8/14	NA	NA	NA
Genetic: *C9orf72/MAPT*	3/6	4/0	NA	NA	NA
Age (years)	64.7 (52–79)	64.9 (52–75)	66.3 (60–73)	66.4 (53–80)	67.5 (54–80)
Education (years)	14 (11–21)	15 (11–20)	13 (11–17)	13.9 (12–17)	15.2 (10–18)
Symptom duration (years)	6.1 (3–18)	5.7 (3–21)	6 (4–9)	4.4 (2–6)	NA
MMSE	**24 (12–30)**	**19 (1–30)**	24 (20–25)	**20 (13–30)**	29.6 (28–30)
**IQ**					
Verbal	**80 (40–119)**	**82 (55–115)**	**94 (71–115)**	**84 (55–108)**	120 (101–137)
Performance	**101 (74–135)**	**96 (66–135)**	**86 (61–125)**	**84 (57–119)**	115 (84–141)
**Episodic memory**					
RMT faces (/50)	**31 (24**–**50)**^a^	**37 (25–46)**	**36 (27–43)**	**32 (23–46)**	43 (30–50)
RMT words (/50)	**34 (23–49)**	**37 (18–48)**	**34 (27–47)**	**31 (27–42)**	48 (39–50)
**Semantic processing**					
BPVS (/150)	**110 (2–149)**	**109 (25–149)**	133 (106–146)	**124 (52–147)**	147 (137–150)
**Executive function**					
Stroop inhibition (180 s)	**88 (40–180)**	**103 (50–180)**	**135 (42–180)**	**107 (73–138)**	58 (35–103)
Digit span reverse (/12)	**4 (0–7)**	**4 (0–7)**	**3.5 (2–6)**	**3 (1–5)**	5 (3–7)
**Visuospatial**					
VOSP object decision (/20)	**16 (8–20)**	**16 (3–20)**	16.5 (14–18)	**16 (11–19)**	18 (12–20)

Mean (range) data are shown unless otherwise indicated and maximum scores on neuropsychology tests are also indicated in parentheses. Significant differences (*p* < .05) between patients and healthy controls are in bold; *historical control group (to reference neuropsychological characterisation of disease groups); †four patients with environmental sound aversion alone, 10 with musicophilia alone, eight with music aversion alone, five with both musicophilia and environmental sound aversion, four with both music and environmental sound aversion (see text, Fig. 1); ††five patients with environmental sound aversion alone, two with both music and environmental sound aversion; ^a^significantly (*p* < .05) different from non-symptomatic patients with FTLD; AD, syndrome of Alzheimer's disease led by decline in episodic memory; BPVS, British Picture Vocabulary Scale; bvFTD, behavioural variant of frontotemporal dementia; *C9orf72*, mutation in open reading frame 72 on chromosome 9; FTLD, frontotemporal lobar degeneration; *MAPT*, mutation in microtubule associated protein tau gene; MMSE, Mini-Mental State Examination score; NA, not applicable; PNFA, progressive nonfluent aphasia; RMT, Recognition Memory Test; SD, semantic dementia; VOSP; Visual Object and Space Perception battery.

**Table 2:** Neuroanatomical associations of auditory hedonic symptoms in the patient cohort.

Auditory hedonic symptom	Brain region	Side	Cluster size (voxels)	Co-ordinates (mm)			z-score
				x	y	z	
**FTLD**							
Any hedonic alteration	Anterior superior temporal gyrus	R	3002	54	15	−9	**4.86**
	Anterior superior temporal sulcus	R		51	5	−15	4.43
Environmental sound aversion	Anterior temporal cortex	R	5275	42	17	−26	**4.88**
	Nucleus accumbens	R	668	3	15	−9	4.24
	Amygdala	L	413	−20	−3	−26	3.66
	Inferior temporal gyrus	R	209	50	0	−39	3.72
Music aversion	Hippocampus/amygdala	R	2864	26	−25	−24	4.69
	Mid – posterior insula	R		44	−1	−12	4.20
	Entorhinal/parahippocampal cortex	R		24	2	−35	4.10
	Mid – posterior insula	L	128	−42	−9	−5	3.83
**AD**							
Environmental sound aversion	Anterior cingulate cortex	L	87	−12	32	31	4.21

All regions of grey matter atrophy shown were significantly associated with auditory hedonic symptoms at threshold *p* < .05_FWE_ corrected for multiple comparisons within the pre-specified anatomical small region of interest; associations in bold were additionally significant at *p* < .05_FWE_ corrected over the whole brain volume. All significant clusters >40 voxels are shown and peak (local maximum) coordinates are in Montreal Neurological Institute standard stereotactic space (see also Fig. 2). AD, Alzheimer's disease; FTLD, frontotemporal lobar degeneration. See text for explanation of symptom classifications.
