# Supplementary material for: Auditory hedonic phenotypes in dementia: A behavioural and neuroanatomical analysis
Source: Cortex. 2015 Jun;67:95–105. doi: 10.1016/j.cortex.2015.03.021 (PMC4465962; doi:10.1016/j.cortex.2015.03.021)
Supplement: Supplementary file 1 [file mmc1.docx]

**SUPPLEMENTARY MATERIAL. Auditory hedonic phenotypes in dementia, by PD Fletcher et al**

**Table S1.** Caregiver questionnaire to assess auditory hedonic symptoms

Has he/she become more sensitive to sound than before the illness?

If yes, please give details:

Does he/she seem to find some sounds more pleasant or less pleasant than before the illness?

If yes, please give details:

Has his/her appreciation of music altered compared with before the illness?

If yes, please give details:

Has his/her liking for sweet foods altered compared with before the illness?

If yes, please give details:

**Table S2.** Representative care-giver comments for patients with auditory hedonic symptoms

| **Case** | **Subgroup** | **Comment** |
| --- | --- | --- |
| 1 | bvFTD:  *C9orf72* | Seems sensitive to loud music. Only listens to music for short periods of time now. |
| 2 |  | More sensitive to any noise. Doesn't like listening to music any more. |
| 3 |  | Increasingly sensitive to noises, in particular children's voices, finds these unpleasant and agitated by them. Listens to i-pod constantly now loves music, plays it very loudly even throughout the night |
| 4 | bvFTD: *MAPT* | Doesn’t really like music any more as it gives no pleasure |
| 5 |  | Plays less music at home, doesn't enjoy it anymore |
| 6 |  | Used to sing in choir and play in orchestra, now never puts on music to listen to at home any more. |
| 7 |  | Does not like any loud noises of any sort, easily agitated by fire alarms, helicopters overhead, sirens |
| 8 |  | Doesn’t like loud bangs or crashes which never used to bother before. |
| 9 |  | Notices sounds from neighbours or planes more than others do, irritated by the sound of furniture scraping on the floor. Listens to music all the time now, same records over and over |
| 10 | bvFTD: sporadic | More sensitive to sound. Used to love classical music but now turns the radio off, even when I am listening, doesn't enjoy music any more |
| 11 |  | Will sit and watch music videos constantly on the music channel for hours at a time |
| 12 |  | Likes music more, has bought a juke box, plays this a lot and has started trying to re-learn the piano! Emotional in response to music |
| 13 |  | Now likes complete silence without radio or TV; children's voices are particularly unpleasant and will sit as far away as possible from the grandchildren when we visit |
| 14 |  | Can’t stand certain sounds, especially the sound of birds tweeting or young children's voices, engine sounds. But really enjoys music, and has started trying to play the piano and sing (loudly) all the time |
| 15 |  | Hearing seems to have been heightened. Constantly buying music CDs, obsessively uploads music to i-pod, plays it very loudly |
| 16 |  | Loud sounds now generally upsetting but plays music very loudly 24 hours a day now. |
| 17 |  | Likes music more |
| 18 |  | Loud sounds are distressing and jumps very easily if there is a sudden loud noise. Background music causes huge irritation and distress |
| 19 |  | Hypersensitive to noise on the train will move away from people who are talking even if it means standing, won’t have the gas fire on (it 'pops'). Demands music on radio turned off even in other room. |
| 20 | SD | No interest in listening to music now, less appreciative of any music. |
| 21 |  | Loves all kinds of music more than before |
| 22 |  | Likes listening to music more, dances along |
| 23 |  | Seems to enjoy loud sounds even if non-musical, the new hand driers give auditory pleasure. More aware of music and gets more emotional |
| 24 |  | Finds loud noises more irritating |
| 25 |  | Real dislike of noises now, even if not particularly loud, especially children's voices, trains, sirens, reduced to tears by sound of a fast train passing through station Likes music more; now obsessed with music videos from 40s and 50s, watches these >50 times a day, has also begun picking out the same tunes on the piano (last played 25 years ago). Obsessional, missed Christmas lunch to do this. |
| 26 |  | Always had the radio or a CD on, now hardly bothers listening to the radio and will never play music |
| 27 |  | Wants to play music more and louder, likes music more now. |
| 28 |  | Listens to music a lot more now |
| 29 |  | Leaves the room if coffee ground or food processor on. Doesn’t enjoy listening to music like before |
| 30 |  | Very upset by loud noises, e.g. passing trains, hairdryers, children's voices. Likes music more - obsessed with particular 1950s singers |
| 31 | PNFA | Does not enjoy listening to music any more though previously enjoyed a wide range |
| 32 | AD | More irritated by noises like TV and radio than before |
| 33 |  | Complains TV and radio too noisy when they are not, finds voices more irritating |
| 34 |  | Does not like the sound of the telephone ringing now |
| 35 |  | Seems more sensitive to loud sounds, finds voices unpleasant |
| 36 |  | Now seems to find Big Ben tolling on the news and similar tones quite excruciating, finds high pitched sounds in films unbearable. Used to enjoy rock music but now finds most music just irritating noise |
| 37 |  | Less tolerant of everyday sounds, finds them more irritating (e.g., some recent not particularly loud building work nearby caused distress) |
| 38 |  | Hearing seems to have become more sensitive especially to high pitched noises like children screaming. Now finds music irritating |

Key: AD, typical syndrome of Alzheimer’s disease; bvFTD, behavioural variant frontotemporal dementia; *C9orf72*, mutation in open reading frame 72 on chromosome 9; FTLD, frontotemporal lobar degeneration; *MAPT*, mutation in microtubule associated protein tau gene; PNFA, progressive nonfluent aphasia; SD, semantic dementia
